# Torus Pairwise Disjoint-Path Routing †

**DOI:** 10.3390/s18113912

**Published:** 2018-11-13

**Authors:** Antoine Bossard, Keiichi Kaneko

**Affiliations:** 1 Graduate School of Science, Kanagawa University, Kanagawa 259-1293, Japan; 2 Graduate School of Engineering, Tokyo University of Agriculture and Technology, Tokyo 184-8588, Japan; k1kaneko@cc.tuat.ac.jp

**Keywords:** parallel processing, interconnect, system dependability, fault tolerance, algorithm

## Abstract

Modern supercomputers include hundreds of thousands of processors and they are thus massively parallel systems. The interconnection network of a system is in charge of mutually connecting these processors. Recently, the torus has become a very popular interconnection network topology. For example, the Fujitsu K, IBM Blue Gene/L, IBM Blue Gene/P, and Cray Titan supercomputers all rely on this topology. The pairwise disjoint-path routing problem in a torus network is addressed in this paper. This fundamental problem consists of the selection of mutually vertex disjoint paths between given vertex pairs. Proposing a solution to this problem has critical implications, such as increased system dependability and more efficient data transfers, and provides concrete implementation of green and sustainable computing as well as security, privacy, and trust, for instance, for the Internet of Things (IoT). Then, the correctness and complexities of the proposed routing algorithm are formally established. Precisely, in an *n*-dimensional *k*-ary torus (n<k, k≥5), the proposed algorithm connects *c* (c≤n) vertex pairs with mutually vertex-disjoint paths of lengths at most 2k(c−1)+n⌊k/2⌋, and the worst-case time complexity of the algorithm is $O(nc^{4})$. Finally, empirical evaluation of the proposed algorithm is conducted in order to inspect its practical behavior.

## 1. Introduction

Since the development of parallel supercomputers, the number of included processors has been continuously rising. The hardware component that is responsible for the connection of processors is the interconnection network (a.k.a., interconnect). Hypercubes [1] were a popular topology for the interconnection network of massively parallel systems in the eighties. Intel developed, for instance, the iPSC supercomputer series, with the iPSC/1 device connecting 32 to 128 cores: the iPSC/d5 is based on a five-dimensional hypercube, hence connecting $2^{5}=32$ cores; the iPSC/6 is based on a six-dimensional hypercube, hence connecting 64 cores, and the iPSC/d7 128 cores. A similar approach was followed by the nCUBE company that built, for example, the nCUBE 10 device, which is based on a 10-dimensional hypercube, hence connecting 1024 cores.

Nowadays, machines of the megaFLOPs era, such as the nCUBE, have been replaced by ones featuring a computing power of several petaFLOPs, and with even the exaFLOP barrier possibly being reached as soon as 2020 by the Cray company [2]. Regarding the number of processors embodied, modern massively parallel systems rely on hundreds of thousands of them. One step ahead, the Sunway TaihuLight supercomputer, ranked world number one in June 2017 and second as of June 2018 [3], includes more than one million cores. Considering this very large number of processors, it is easy to understand that data communication efficiency and interconnection networks in general are critical to achieving the highest computing performance. Indeed, in the case of suboptimal core interconnection and data transfers, bottleneck situations would inevitably arise, inducing underused cores.

Just as complete networks are not practical as interconnection networks of massively parallel systems given the prohibitively high number of edges per processor induced, hypercubes are no more a solution considering the number of nodes involved. Precisely, the vertex degree in the case of a hypercube becomes rapidly impractical as *n* edges per vertex are required for the hypercube to connect, in total, $2^{n}$ vertices. As a result, various topologies designed for interconnection networks have been introduced. For example, Li et al. proposed the dual-cube [4] and metacube [5] topologies, both based on hypercubes. The star-graph [6] is another topology example, this time based on permutations, which was used to introduce other topologies, such as burnt pancake graphs [7].

Topologies based on meshes [8] are another solution for interconnection networks. One of these, the torus topology [9], is addressed in this paper. Thanks to a simple definition and an advantageous network order compared with, for instance, hypercubes, the torus network topology is very popular as interconnect of supercomputers. Indeed, numerous machines listed in the TOP500 world ranking are based on the torus topology. For example, the Fujitsu K (Tofu interconnect [10,11]), IBM Blue Gene/L, IBM Blue Gene/P, and Cray Titan (Gemini interconnect [12]) supercomputers [3]. The main topological properties of tori and those of various other interconnection networks are given in Table 1. In this table, the network cost is the product of network degree by network diameter.

In this paper, the torus pairwise disjoint-path routing problem is addressed. This critical data communication problem consists of the selection of mutually vertex-disjoint paths between several vertex pairs. As detailed next, this problem has numerous applications. Disjoint-path routing in general is a very desirable property for routing algorithms, and this is for several reasons. First, disjoint-path routing enables simultaneous data transfers over the network, and for that, without the need to change switching patterns inside routers, on the one hand optimizing data transfer performance, and, on the other hand, reducing network usage time. While the former consequence has obvious positive implications, the latter thus induces concrete implementation of energy-harvesting communications and networks, which contents green and sustainable computing [13,14], with applications, for instance, to the Internet of Things (IoT) and cyber–physical systems. In addition, path disjointness enforced at the hardware level with circuit switching means that an optimum degree of privacy is achieved as a data transfer is never interrupted by another transmission. This research thus directly addresses the issues of security, privacy, and trust for the IoT. Second, disjoint-path routing importantly ensures that the notorious blocking situations of parallel processing, deadlocks, livelocks and starvations, never occur, thus facilitating, for instance, distributed data processing in sensor applications. Third, not only efficiency but, by selecting disjoint paths, system dependability is also significantly increased. Effectively, where multiple nondisjoint paths could be rendered useless by a single faulty vertex (i.e., the faulty vertex is common to several paths), disjoint paths are much more robust: one faulty vertex can jeopardize, at most, one path, since any vertex of the network is included in at most one of the selected paths. Such robustness has positive implications regarding, for instance, the quality of experience and service in the IoT and cyber–physical systems.

In an arbitrary *c*-connected graph G(V,E), the existence of *c* disjoint paths for each of the node-to-node disjoint paths, the node-to-set disjoint paths, and the set-to-set disjoint paths problems are ensured by Menger’s theorem [15]. In fact, disjoint paths can be obtained by applying the maximum-flow algorithm assigning unit capacity to each of the edges and the vertices. For *c* pairs of vertices in arbitrary graph G(V,E), the problem whether there are *c* disjoint paths between the *c* pairs is NP complete if *c* is a variable of the problem input [16]. For any fixed *c*, the problem can be solved in $O(|V{|}^{3})$ [17] though it is still a hard problem.

Due to higher complexity, the pairwise disjoint-path routing problem is typically addressed last after introducing algorithms that solve the unicast (a.k.a., point-to-point, one-to-one, or node-to-node), node-to-node disjoint-path, node-to-set disjoint-path and set-to-set disjoint-path routing problems. In an *n*-dimensional *k*-ary torus, a node-to-node routing algorithm has been described in Reference [18] and with fault tolerance in Reference [19], the latter selecting paths of lengths at most n⌊k/2⌋+1, with a worst-case time complexity of $O(k^{2})$. A node-to-set disjoint-path routing algorithm in a torus has also been described in Reference [19], with paths of lengths at most n⌊k/2⌋+1, and a worst-case time complexity of $O(k^{3})$. A torus set-to-set disjoint-path routing algorithm has been given in Reference [20], with paths of lengths at most 2(k+1)n, and a worst-case time complexity of $O(kn^{3}+n^{3}\log n)$.

As for different topologies, Gu and Peng described a pairwise disjoint-path routing algorithm in a hypercube [21] and in a star-graph [22]. Bossard and Kaneko solved the same problem in perfect hierarchical hypercubes [23], as did Sawada et al. in pancake graphs [24], and Park focused on restricted hypercube-like graphs [25]. The approach in this paper to select mutually vertex-disjoint paths is, as used in several previous works, to rely on the recursive structure of a torus. Precisely, in an *n*-dimensional *k*-ary torus, given *c* (c≤n) vertex pairs, the algorithm proposed here selects *c* mutually vertex-disjoint paths of lengths at most 2k(c−1)+n⌊k/2⌋ with a worst-case time complexity of $O(nc^{4})$.

Related research on disjoint-path routing can be found in Reference [26] where it is applied to data-center networks. Close to disjoint-path routing, independent spanning tree construction has been researched, for instance, in Möbius cubes in [27]. Other related works include the calculation of various topological values of the network topology, such as topological indices [28] and the least eigenvalue [29].

The rest of this paper is organized as follows. Definitions, notations and intermediary results are established in Section 2. The proposed routing algorithm is described in Section 3 and exemplified in Section 4. The proof of the algorithm correctness, including the fact that the selected paths are disjoint, is given in Section 5, which relies on lemmas proved in Section 2. Complexities are formally established in Section 6 and the proposed algorithm is empirically verified and evaluated in Section 7. Finally, this paper is concluded in Section 8.

## 2. Preliminaries

For the sake of clarity, a vertex pair (in this paper, typically a source-destination vertex pair), denoted by (u,v), can be considered as the set {u,v}. For vertex *u*, define set N(u) as the set of the neighbor vertices of *u*.

In a graph, a path *p* is an alternate sequence of vertices and links $u_{1},\left(u_{1},u_{2}\right),u_{2},\ldots ,\left(u_{n-1},u_{n}\right),u_{n}$. Path *p* can be similarly written as $u_{1}\to u_{2}\to \ldots \to u_{n}$ and abbreviated as $u_{1}⇝u_{n}$ when explicit mention of the vertices between $u_{1}$ and $u_{n}$ is not required. The length of a path is defined as its number of links, hence *p* has length n−1. Two paths are mutually vertex-disjoint (simply *disjoint* hereinafter) if and only if they have no vertex in common.

**Definition** **1** **([8]).** *An n-dimensional k-ary torus, denoted by (n, k)-torus, is an undirected graph made of the $k^{n}$ vertices induced by the set ${\left\{0,1,\ldots ,k-1\right\}}^{n}$. Two vertices $a=(a_{1},a_{2},\ldots ,a_{n})$ and $b=(b_{1},b_{2},\ldots ,b_{n})$ of an (n, k)-torus are adjacent if and only if $\exists j\;\left(1\leq j\leq n\right),\forall i\;\left(1\leq i\leq n,i\neq j\right),a_{i}=b_{i},a_{j}=\left(b_{j}\pm 1\right)\;\mathrm{mod}\;k$.*

**Figure 1:**
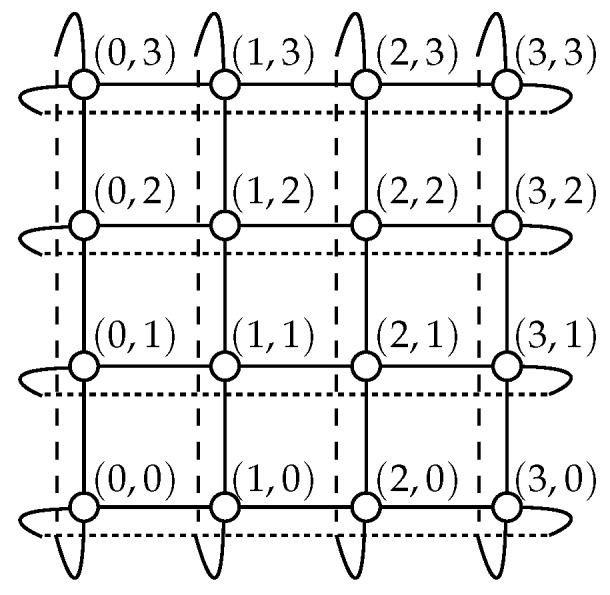
A two-dimensional four-ary torus, with vertex addresses in the top right-hand corner of each vertex [9].

Thus, a vertex of an (*n*, *k*)-torus is an *n*-dimensional vector. Therefore, two vertices of an (*n*, *k*)-torus can be compared (i.e., equality testing) in linear O(n) time, by simply going through each of all the *n* coordinates of the two vertices. A (2, 4)-torus is illustrated in Figure 1.

An (*n*, *k*)-torus is a recursive topology: for one dimension δ (1≤δ≤n), it consists of *k* (n−1, *k*)-tori (called *subtori*). For instance, considering the horizontal dimension of the (2, 4)-torus of Figure 1, that is the dimension δ=1, this torus consists of four (1, 4)-tori: these subtori appear vertically in the figure. It is said that a path goes through a subtorus *T* if and only if it includes a vertex of *T*.

Next, operator γ is defined for convenient vertex coordinate manipulation. In an (*n*, *k*)-torus, for a vertex $u=(u_{1},u_{2},\ldots ,u_{n})$ and a dimension δ (1≤δ≤n), let γ(u,δ) denote the δ coordinate of vertex *u*. For instance, $\gamma \left(u,4\right)=u_{4}$. This definition is extended to subtori: let γ(T,δ) denote the δ coordinate of subtorus *T*. For instance, considering the torus of Figure 1 and the dimension δ=1, we have γ(T,δ)=0 where *T* is the leftmost subtorus (i.e., *T* consists of the vertices (0,0), (0,1), (0,2) and (0,3)). Therefore, for dimension δ, a subtorus *T* can be unambiguously specified by the value of γ(T,δ).

Then, still considering vertex *u* and dimension δ, candidate paths and the corresponding path sets for traversing the dimension δ are defined as follows. Note that these paths specify how to traverse a dimension, thus remaining “open” (i.e., with a start vertex, but no end vertex) for the sake of simplicity.

**Definition** **2.** *Given two vertices $a=(a_{1},a_{2},\ldots ,a_{n})$ and $b=(b_{1},b_{2},\ldots ,b_{n})$ of an (n, k)-torus, define $a⊕b=(\left(a_{1}+b_{1}\right)\;\mathrm{mod}\;k,\left(a_{2}+b_{2}\right)\;\mathrm{mod}\;k,\ldots ,\left(a_{n}+b_{n}\right)\;\mathrm{mod}\;k)$ and $a⊖b=(\left(a_{1}-b_{1}\right)\;\mathrm{mod}\;k,\left(a_{2}-b_{2}\right)\;\mathrm{mod}\;k,\ldots ,\left(a_{n}-b_{n}\right)\;\mathrm{mod}\;k)$.*

**Definition** **3.** *n unit vectors $e_{i}$ (1≤i≤n) are defined as $\left(u_{1},u_{2},\ldots ,u_{n}\right)$ where $u_{j}=0$ (1≤j≤n, i≠j) and $u_{i}=1$.*

First, define the path:

$$p^{+}\left(u,\delta \right):\;u\to u⊕e_{\delta }\to \left(u⊕e_{\delta }\right)⊕e_{\delta }\to \ldots$$

which can be similarly written as $u\to \left(u_{1},u_{2},\ldots ,\left(u_{\delta }+1\right)\;\mathrm{mod}\;k,\ldots ,u_{n}\right)\to \left(u_{1},u_{2},\ldots ,\left(u_{\delta }+2\right)\;\mathrm{mod}\;k,\ldots ,u_{n}\right)\to \ldots$

Next, define the two path sets:

$$\begin{array}{c} P_{1}^{+}\left(u,\delta \right):\;{⋃}_{\begin{array}{c} 1\leq i\leq n\\ i\neq \delta \end{array}}\left\{\begin{array}{c} u\to u⊕e_{i}\to \left(u⊕e_{i}\right)⊕e_{\delta }\to \left(\left(u⊕e_{i}\right)⊕e_{\delta }\right)⊕e_{\delta }\to \ldots ,\\ u\to u⊖e_{i}\to \left(u⊖e_{i}\right)⊕e_{\delta }\to \left(\left(u⊖e_{i}\right)⊕e_{\delta }\right)⊕e_{\delta }\to \ldots \end{array}\right\} \end{array}$$

and

$$\begin{array}{c} P_{2}^{+}\left(u,\delta \right):\;{⋃}_{\begin{array}{c} 1\leq i\leq n\\ i\neq \delta \end{array}}\left\{\begin{array}{c} u\to u⊕e_{i}\to \left(u⊕e_{i}\right)⊕e_{i}\to \left(\left(u⊕e_{i}\right)⊕e_{i}\right)⊕e_{\delta }\\ \;\to \left(\left(\left(u⊕e_{i}\right)⊕e_{i}\right)⊕e_{\delta }\right)⊕e_{\delta }\to \ldots ,\\ u\to u⊖e_{i}\to \left(u⊖e_{i}\right)⊖e_{i}\to \left(\left(u⊖e_{i}\right)⊖e_{i}\right)⊕e_{\delta }\\ \;\to \left(\left(\left(u⊖e_{i}\right)⊖e_{i}\right)⊕e_{\delta }\right)⊕e_{\delta }\to \ldots \end{array}\right\} \end{array}$$

Path $p^{-}\left(u,\delta \right)$ and path sets $P_{1}^{-}\left(u,\delta \right)$, $P_{2}^{-}\left(u,\delta \right)$ are defined similarly but with $⊖\;e_{\delta }$ instead of $⊕\;e_{\delta }$ (details in the appendix). In other words, path $p^{+}\left(u,\delta \right)$ (resp. $p^{-}\left(u,\delta \right)$) makes no detour when traversing dimension δ, while the paths of $P_{1}^{+}\left(u,\delta \right)$ and $P_{2}^{+}\left(u,\delta \right)$ (resp. $P_{1}^{-}\left(u,\delta \right)$ and $P_{2}^{-}\left(u,\delta \right)$) make a detour of one and two links, respectively.

Finally, define the two path sets:

$$P^{+}\left(u,\delta \right)=\left\{p^{+}\left(u,\delta \right)\right\}\cup P_{1}^{+}\left(u,\delta \right)\cup P_{2}^{+}\left(u,\delta \right)$$

and

$$P^{-}\left(u,\delta \right)=\left\{p^{-}\left(u,\delta \right)\right\}\cup P_{1}^{-}\left(u,\delta \right)\cup P_{2}^{-}\left(u,\delta \right)$$

**Figure 2:**
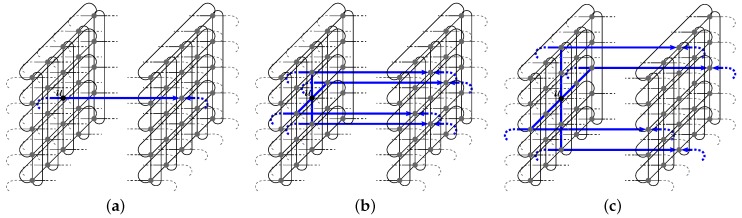
Considered paths (represented by arrows) for traversing the subtori of a (3, 5)-torus from the vertex *u*. (**a**) $p^{-}\left(u,\delta \right)$, $p^{+}\left(u,\delta \right)$, (**b**) $P_{1}^{-}\left(u,\delta \right)$, $P_{1}^{+}\left(u,\delta \right)$ and (**c**) $P_{2}^{-}\left(u,\delta \right)$, $P_{2}^{+}\left(u,\delta \right)$.

The paths of $P^{+}\left(u,\delta \right)$ and $P^{-}\left(u,\delta \right)$ in the case of a (3, 5)-torus are illustrated in Figure 2, where δ is the dimension used to distinguish subtori.

For the sake of conciseness, for torus vertex *u* and dimension δ, paths $p^{+}\left(u,\delta \right)$ and $p^{-}\left(u,\delta \right)$, as well as path sets $P_{1}^{+}\left(u,\delta \right)$, $P_{1}^{-}\left(u,\delta \right)$, $P_{2}^{+}\left(u,\delta \right)$ and $P_{2}^{-}\left(u,\delta \right)$ are abbreviated to p(u,δ), $P_{1}\left(u,\delta \right)$ and $P_{2}\left(u,\delta \right)$, respectively, when no ambiguity arises.

Next, a torus point-to-point routing algorithm is recalled. Unicast routing in a torus can be achieved simply with a dimension-order routing algorithm. A dimension-order routing algorithm for packet forwarding in two dimensional meshes is presented by Duato et al. [8]. It can be easily adjusted for routing in an *n*-dimensional torus as listed in Algorithm 1. The maximum length of a path selected by this algorithm between any two vertices is thus n⌊k/2⌋.

To conclude this section, we introduce two essential lemmas on which the algorithm proposed in this paper is based (Section 3).

**Lemma** **1.** *In an (n, k)-torus (n≥2, k≥5), given c≤n vertex pairs $\left(s_{i},d_{i}\right)$ satisfying $\left\{s_{i},d_{i}\right\}\cap \left\{s_{j},d_{j}\right\}=\emptyset$ (1≤i,j≤c, i≠j) with thus $s_{i}=d_{i}$ allowed, and one subtorus T on a dimension δ (1≤δ≤n), disjoint paths (at the exception that the paths for $s_{i}$ and $d_{i}$ (1≤i≤c) need not be disjoint) that route each of all vertices $s_{i},d_{i}$ to T can be found in $O(nc^{2})$ time. Maximum path length is k+1.*

**Algorithm 1:** Point-to-point simple routing in a torus with a dimension-order routing algorithm. 
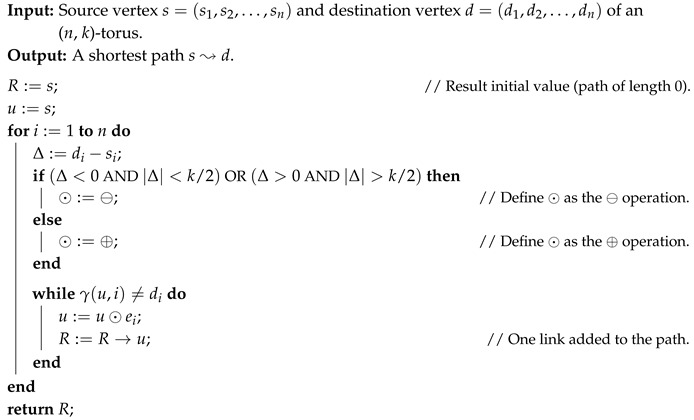

**Proof.**  First, assume without loss of generality that $s_{i}$ is to be routed to *T*, and that $\gamma \left(s_{i},\delta \right)\leq \gamma \left(T,\delta \right)$. The same discussion holds for $d_{i}$ and for the case $\gamma \left(s_{i},\delta \right)>\gamma \left(T,\delta \right)$. We show that there always remains at least one path of $P^{+}\left(s_{i},\delta \right)$ that is not blocked by another source vertex, destination vertex or their respective paths towards *T*.

**Figure 3:**
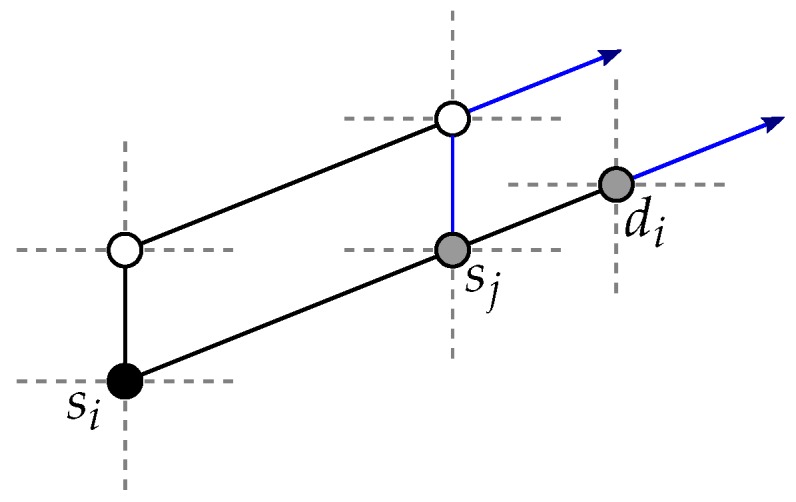
Vertex $d_{i}$ can be indirectly a blocker for vertex $s_{i}$ (selected paths in blue) [9].

First, the paths of $P^{+}\left(s_{i},\delta \right)$ consist of shortest path $p^{+}\left(s_{i},\delta \right)$, and of the two sets $P_{1}^{+}\left(s_{i},\delta \right)$ and $P_{2}^{+}\left(s_{i},\delta \right)$. The paths of these two sets are not disjoint: a neighbor *u* of $s_{i}$ is included in one single path of each of the two path sets. Hence, if $u=s_{j}$ (i≠j), two candidate paths are blocked by one single vertex ($s_{j}$). The reason for considering $P_{1}$ and $P_{2}$ paths is that vertex $d_{i}$ can indirectly block a candidate path for $s_{i}$. Effectively, $d_{i}$ may trigger the selection of a non-shortest path for a vertex $s_{j}$ towards *T* (i≠j), and thus in total $d_{i}$ and $s_{j}$ each block one path for $s_{i}$. See Figure 3.

Vertex $d_{i}$ on its own is not a blocker for $s_{i}$. Thus, we consider the case where $d_{i}$ is an indirect blocker through vertex say $s_{j}$.

So, by considering the paths of $P_{1}$ and $P_{2}$, we ensure that either there remains one of the two paths, $\left(s_{i}\to u\to \ldots \right)\in P_{1}^{+}\left(s_{i},\delta \right)$ and $\left(s_{i}\to u\to \ldots \right)\in P_{2}^{+}\left(s_{i},\delta \right)$ that is not blocked by $d_{i},s_{j}$, or, if not, $d_{i},s_{j}$ block two nondisjoint paths of $P_{1}^{+}\left(s_{i},\delta \right)\cup P_{2}^{+}\left(s_{i},\delta \right)$. In the first situation, we necessarily have $s_{j},d_{i}\notin N\left(s_{i}\right)$, thus $p^{+}\left(s_{i},\delta \right)$ and one path of $P_{1}^{+}\left(s_{i},\delta \right)$ are blocked *but* the path $s_{i}\to u\to \ldots$ of $P_{2}^{+}\left(s_{i},\delta \right)$ remains unblocked. In the second situation, two nondisjoint paths are blocked by $d_{i},s_{j}$; thus, $d_{i},s_{j}$ count as one blocker for $s_{i}$ (they block only one of the *disjoint* candidate paths). Therefore, it is sound to assume that $d_{i}$ does not count as a blocker for $s_{i}$.

**Figure 4:**
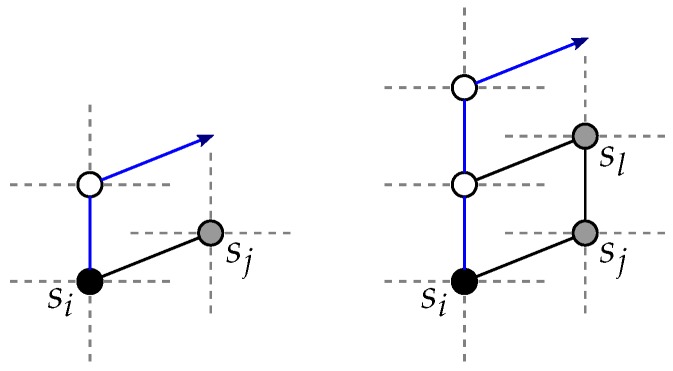
A vertex of $\left(S\cup D\right)\setminus \left\{s_{i},d_{i}\right\}$ can block at most one of the disjoint candidate paths for a vertex $s_{i}$ (selected paths in blue) [9].

At the exception of $d_{i}$, each of all 2(n−1) other blockers for $s_{i}$ (i.e., $\left(S\cup D\right)\setminus \left\{s_{i},d_{i}\right\}$) can block at most one of the disjoint candidate paths. In total, it is possible to select 2(n−1)+1 disjoint paths from $s_{i}$ to *T* (i.e., considering the candidate paths $\left\{p^{+}\left(s_{i},\delta \right)\right\}\cup P_{1}^{+}\left(s_{i},\delta \right)\cup P_{2}^{+}\left(s_{i},\delta \right)$). Therefore, considering that at most 2(n−1) of these disjoint paths are blocked, there always remain [2(n−1)+1]−2(n−1)=1 path to route $s_{i}$ to *T*. See Figure 4.

Maximum path length would be obtained if $s_{i}$ is routed to *T* with a path of $P_{2}$, hence of length (k−1)+2=k+1. To route $s_{i}$ to *T*, we first enumerate the paths of $\left\{p^{+}\left(s_{i},\delta \right)\right\}\cup P_{1}^{+}\left(s_{i},\delta \right)$ (starting with $p^{+}\left(s_{i},\delta \right)$). If all are blocked, it means that $d_{i}$ is an (indirect) blocker, thus inducing the check of at most one path of $P_{2}$ (i.e., if the first checked path of $P_{2}$ is blocked, the second one will always do). Hence, the worst-case time complexity to route $s_{i}$ to *T* is O(nc), and, in total, $O(nc^{2})$ is required to route all vertices $s_{i},d_{i}$ (1≤i≤c) to *T*. ☐

**Lemma** **2.** *In an (n, k)-torus (n≥3, k≥5), given 3≤c≤n vertex pairs $\left(s_{i},d_{i}\right)$ satisfying $\left\{s_{i},d_{i}\right\}\cap \left\{s_{j},d_{j}\right\}=\emptyset$ (1≤i,j≤c, i≠j) with thus $s_{i}=d_{i}$ allowed, and two subtori $T,T^{\prime }$ on a dimension δ (1≤δ≤n), disjoint paths (at the exception that the paths for $s_{i}$ and $d_{i}$ (1≤i≤c) need not be disjoint) that route the vertices of one pair to $T^{\prime }$ without going through T and the vertices of the other pairs to T without going through $T^{\prime }$ can be found in $O(nc^{2})$ time. Maximum path length is k.*

**Figure 5:**
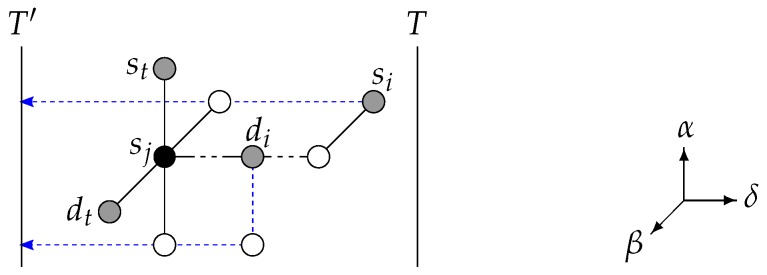
Illustration of the case n=c=3 with the pair $\left(s_{i},d_{i}\right)$ blocking 3 paths for the pair $\left(s_{j},d_{j}\right)$ when routed to the subtorus $T^{\prime }$. Vertex $s_{j}$ is not routable to subtorus *T* without going through $T^{\prime }$. (Dimension names are given on the right for reference.)

**Proof.**  Assume that, given the pair $\left(s_{i},d_{i}\right)$ routed to $T^{\prime }$, there exists vertex $s_{j}$ (i≠j) that cannot be disjointly routed to *T* without going through $T^{\prime }$, that is, each of all candidate paths to *T* for $s_{j}$ as per Lemma 1 are blocked. See Figure 5. If such a vertex $s_{j}$ does not exist, all vertices $s_{t},d_{t}$ (1≤t≤c, t≠i) can be routed to *T* provided that if there exists a unique pair $\left(s_{j},d_{j}\right)$ (j≠i) with 3 disjoint candidate paths to *T* without going through $T^{\prime }$ blocked by the paths for $\left(s_{i},d_{i}\right)$ it is routed first to *T*, (i.e., before all the other pairs $\left(s_{t},d_{t}\right)$ (1≤t≤c, t≠i,t≠j)), and there is thus nothing to prove.

For $s_{j}$ to be fully blocked, vertex $s_{i}$ or $d_{i}$ (i≠j) needs to be on dimension δ, that is, blocking path $p(s_{j},\delta )$. By definition of the candidate paths (see Lemma 1), this vertex $s_{i}$ or $d_{i}$ is the only one that can block two candidate paths for $s_{j}$, as all the other vertices $s_{l},d_{l}$ (l≠j,l≠i) can block, at most, one candidate path for $s_{j}$. Hence, the 2(n−1)+1 candidate paths for $s_{j}$ are blocked by at least 2(n−1) vertices $s_{i}$ or $d_{i}$ (i≠j). In other words, for vertex $s_{j}$ to be fully blocked, vertex $s_{i}$ or $d_{i}$ *needs* to be on $p(s_{j},\delta )$, the unique direct path to *T* for $s_{j}$. Since at least 2(n−1) vertices $s_{i}$ or $d_{i}$ are required to fully block $s_{j}$, it means that one of these 2(n−1) blockers blocks $p(s_{j},\delta )$ in addition to one of the two candidate paths for $s_{j}$ on another dimension, say α (α≠δ). All other 2(n−1)−1 blockers are positioned 2 per dimension β (β≠δ,β≠α), at the exception of one blocker that is on the dimension α (on the other side of $s_{j}$ compared with the blocker that blocks two paths at once for $s_{j}$). Hence, there exists one unique such vertex of S∪D (here $s_{j}$) that is not routable to *T*.

So, for a vertex $s_{j}$ to be fully blocked, T∩(S∪D)=∅ is induced, otherwise it would mean that at least one pair vertex needs not be routed at all (i.e., a path of zero length will do for that vertex), and, thus, all other vertices can be routed to $T,T^{\prime }$ by Lemma 1.

**Figure 6:**
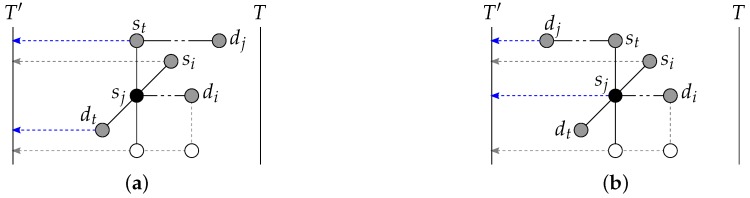
Illustration of the case n=c=3 with the pair $\left(s_{i},d_{i}\right)$ blocking 3 paths for the pair $\left(s_{j},d_{j}\right)$ when routed to the subtorus $T^{\prime }$. Vertex $s_{j}$ is not routable to the subtorus *T*. (**a**) Pair $\left(s_{t},d_{t}\right)$ is routed to $T^{\prime }$ in place of $\left(s_{i},d_{i}\right)$. (**b**) Pair $\left(s_{t},d_{t}\right)$ is not routable to $T^{\prime }$ (because of $d_{j}$), so pair $\left(s_{j},d_{j}\right)$ is routed to $T^{\prime }$ in place of $\left(s_{i},d_{i}\right)$.

Furthermore, since c≥3, either there exists a pair $\left(s_{t},d_{t}\right)$ with $\left\{s_{t},d_{t}\right\}\subset N\left(s_{j}\right)$ that can be routed to $T^{\prime }$ without going through *T* (in place of the pair $\left(s_{i},d_{i}\right)$) with the paths $p(s_{t},\delta )$ and $p(d_{t},\delta )$ (see Figure 6a). Or, if $d_{j}$ blocks one of these two paths, the pair $\left(s_{j},d_{j}\right)$ can be routed to $T^{\prime }$ without going through *T* (in place of the pair $\left(s_{i},d_{i}\right)$) with the paths $p(s_{j},\delta )$ and $p(d_{j},\delta )$ (see Figure 6b). Because of the uniqueness of such a vertex $s_{j}$ not routable to *T*, we have shown that it is possible to disjointly route one pair to $T^{\prime }$ and the other pairs to *T*.

Considering the two subtori *T* and $T^{\prime }$, the value of $|\gamma \left(T,\delta \right)-\gamma \left(T^{\prime },\delta \right)|$ is at most k−1. Given that the pair vertices are to be routed towards *T* (resp. $T^{\prime }$) not going through $T^{\prime }$ (resp. *T*), and by Lemma 1, the maximum path length is (k−2)+2=k.

Regarding time complexity, pair vertices can be routed to *T* and $T^{\prime }$ as follows. Let $\left(s_{i},d_{i}\right)$ be the pair routed to $T^{\prime }$. By Lemma 1, this takes O(nc) time. If either $s_{i}$ or $d_{i}$ blocks a path p(u,δ) for *u* a vertex of a pair $\left(s_{j},d_{j}\right)$ (1≤j≤c, j≠i), do as follows, and otherwise each of all pairs except the one routed to $T^{\prime }$ is routed to *T* with a path as per Lemma 1 that does not go through $T^{\prime }$. Check if *u* is routable to *T* with a path as per Lemma 1 that does not go through $T^{\prime }$; this takes O(nc) time. If *u* is routable to *T*, starting with the pair $\left(s_{j},d_{j}\right)$, each of all pairs except $\left(s_{i},d_{i}\right)$ is routed to *T* with a path as per Lemma 1 that does not go through $T^{\prime }$. If *u* not routable to *T*, discard the paths for $\left(s_{i},d_{i}\right)$ to $T^{\prime }$ and instead route an arbitrary pair $\left(s_{t},d_{t}\right)$ (t≠i,t≠j) to $T^{\prime }$ with the paths $p\left(s_{t},\delta \right),p\left(d_{t},\delta \right)$ not going through *T*, with the possibility that $d_{j}$ blocks $\left(s_{t},d_{t}\right)$ when routed to $T^{\prime }$, in which case it is the pair $\left(s_{j},d_{j}\right)$ that is routed to $T^{\prime }$, with the paths $p\left(s_{j},\delta \right),p\left(d_{j},\delta \right)$ not going through *T*. Each of all pairs except the one routed to $T^{\prime }$ is routed to *T* with a path as per Lemma 1 that does not go through $T^{\prime }$. Hence, the induced total time complexity is that of Lemma 1: $O(nc^{2})$. ☐

## 3. Routing Algorithm

In an (*n*, *k*)-torus (n<k, k≥5), given *c* pairs $\left(s_{i},d_{i}\right)$ (1≤i≤c≤n) satisfying $\left\{s_{i},d_{i}\right\}\cap \left\{s_{j},d_{j}\right\}=\emptyset$ (1≤i,j≤c, i≠j); thus, $s_{i}=d_{i}$ allowed, this problem consists in finding a path connecting each $\left(s_{i},d_{i}\right)$ pair (1≤i≤c), and such that the selected *c* paths are mutually vertex-disjoint. The algorithm execution trace of a sample instance of the pairwise routing problem is given in Section 4.

First, one should note that a torus of dimension 1 (i.e., n=1) is isomorphic to a ring. Hence, in this case, c=1, and it is trivial to connect the unique source-destination vertex pair by traversing the ring. So, we henceforth assume that n≥2. The main idea of the proposed routing algorithm is to follow a divide-and-conquer approach by considering the (*n*, *k*)-torus as a *k*-set of (n−1, *k*)-tori, connecting one source-destination pair in one such subtorus, and solving the problem recursively for the remaining pairs in another, distinct subtorus. We proceed according to the following cases.

### 3.1. Base Case: c=1

This is point-to-point routing in a torus, and thus dimension-order routing can be applied to find a shortest path $s_{1}⇝d_{1}$.

### 3.2. General Case: n≥3

**Step** **1** Find a subtorus $T^{\prime }$ such that the following three conditions hold:
  $\forall \gamma =\left\{s_{i},d_{j}\right\}\in S\times D,i\neq j,\;\gamma ⊄T^{\prime }$
  $\forall \gamma^{\prime }=\left\{s_{i},s_{j}\right\}\in S^{2},i\neq j,\;\gamma^{\prime }⊄T^{\prime }$
  $\forall \gamma^{\prime \prime }=\left\{d_{i},d_{j}\right\}\in D^{2},i\neq j,\;\gamma^{\prime \prime }⊄T^{\prime }$
  In other words, subtorus $T^{\prime }$ either includes no source vertex and no destination vertex, includes at most one source vertex and no destination vertex, includes at most one destination vertex and no source vertex, or includes one single source vertex, say $s_{i}$, and one single destination vertex $d_{i}$.
  The selection of subtorus $T^{\prime }$ sets dimension δ to reduce the original (*n*, *k*)-torus to a set of *k* (n−1, *k*)-subtori.
**Step** **2** First, source-destination pair ρ is selected as follows. If $T^{\prime }\cap \left(S\cup D\right)=\emptyset$, any pair will do, so select one arbitrarily, say $\rho =(s_{i},d_{i})$ (1≤i≤c). If $\exists i\;\left(1\leq i\leq c\right),T^{\prime }\cap \left(S\cup D\right)=\left\{s_{i}\right\}$, let $\rho =(s_{i},d_{i})$. Otherwise, that is $\exists i\;\left(1\leq i\leq c\right),T^{\prime }\cap \left(S\cup D\right)=\left\{d_{i}\right\}$, let $\rho =(s_{i},d_{i})$. In the remaining of this section, assume without loss of generality that $\rho =(s_{i},d_{i})$, in other words that the source-destination pair to be connected inside $T^{\prime }$ is $\left(s_{i},d_{i}\right)$.
  Second, considering selected pair $\rho =(s_{i},d_{i})$, select subtorus *T* distinct from $T^{\prime }$, such that ρ∩T=∅.
**Step** **3** Route one source-destination pair to $T^{\prime }$ and the other pairs towards *T* as per Lemma 2.
**Step** **4** If for the pair $\left(s_{i},d_{i}\right)$ that is routed to $T^{\prime }$, say with the paths $p_{s}:s_{i}⇝s_{i}^{\prime }\in T^{\prime }$ and $p_{d}:d_{i}⇝d_{i}^{\prime }\in T^{\prime }$, we have U≠∅ with $U=\left(p_{s}\cap p_{d}\right)\setminus T^{\prime }$, consider the vertex u∈U that is the closest to $s_{i}$. Select the subpath $s_{i}⇝u$ of $p_{s}$ and the subpath $d_{i}⇝u$ of $p_{d}$, and discard the subpath $u⇝s_{i}^{\prime }$ of $p_{s}$ and the subpath $u⇝d_{i}^{\prime }$ of $p_{d}$. Otherwise, apply the algorithm recursively in $T^{\prime }$ to connect $s_{i}$ to $d_{i}$.
**Step** **5** For each pair $\left(s_{j},d_{j}\right)$ routed to *T*, say with the paths $p_{s}:s_{j}⇝s_{j}^{\prime }\in T$ and $p_{d}:d_{j}⇝d_{j}^{\prime }\in T$, such that U≠∅ with $U=(p_{s}\cap p_{d})\setminus T$, consider the vertex u∈U that is the closest to $s_{j}$. Select the subpath $s_{j}⇝u$ of $p_{s}$ and the subpath $d_{j}⇝u$ of $p_{d}$, and discard the subpath $u⇝s_{j}^{\prime }$ of $p_{s}$ and the subpath $u⇝d_{j}^{\prime }$ of $p_{d}$. Define *E* the set of all such source-destination pairs.
**Step** **6** For each source-destination pair not in *E*, apply the algorithm recursively in *T*.

**Figure 7:**
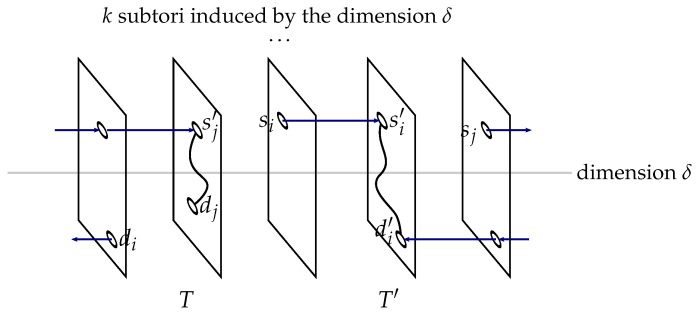
Disjointly connecting one source-destination pair inside subtorus $T^{\prime }$ and the other pairs inside subtorus *T* [9].

An illustration of the algorithm general case is given in Figure 7.

### 3.3. Special Case: n=2 and c=2

Select subtorus $T^{\prime }$ as in Step 1 of the general case, and deduce a pair ρ as in Step 2 of the general case. Route pair ρ to $T^{\prime }$ without going through *T*, and the other pair to *T* with at most one possible path going through $T^{\prime }$ (this may require exchanging the roles of *T* and $T^{\prime }$ as explained in the proof of correctness below).

For each of the two pairs $\left(s_{i},d_{i}\right)$ (1≤i≤2), if the selected paths $p_{s}:s_{i}⇝s_{i}^{\prime }\in T^{\prime }\cup T$ and $p_{d}:d_{i}⇝d_{i}^{\prime }\in T^{\prime }\cup T$ are not disjoint, that is both include a same vertex *u*, discard the subpath $u⇝s_{i}^{\prime }$ of $p_{s}$ and the subpath $u⇝d_{i}^{\prime }$ of $p_{d}$. Otherwise, complete the connection of $s_{i}$ to $d_{i}$ by traversing the subtorus of $s_{i}^{\prime },d_{i}^{\prime }$. The subtori $T^{\prime },T$ are 1-dimensional and thus isomorphic to a ring. It is thus trivial to connect $s_{i}^{\prime },d_{i}^{\prime }$, possibly avoiding one vertex.

## 4. Execution Trace Example

In this section, considering the following disjoint pairwise routing problem instance, a possible execution trace of the proposed routing algorithm is detailed. In a (4, 5)-torus, let $S=\{s_{1}=\left(2,1,0,4\right),$$s_{2}=\left(0,2,1,2\right),s_{3}=\left(2,4,0,2\right),s_{4}=\left(4,4,4,1\right)\}$, $D=\{d_{1}=\left(0,0,4,4\right),$$d_{2}=\left(3,2,0,2\right),$$d_{3}=\left(0,4,0,3\right),$$d_{4}=\left(0,4,0,2\right)\}$ and $\left\{\left(s_{1},d_{1}\right),\left(s_{2},d_{2}\right),\left(s_{3},d_{3}\right),\left(s_{4},d_{4}\right)\right\}$ be the set of vertex pairs to be disjointly connected. Algorithm steps such as subtorus and subpath selection are given in Table 2. The torus arity, here k=5, never changes and does thus not appear in the table.

## 5. Proof of Correctness

First, dimension-order routing is applied in the base case of the recursion when there is one single source-destination pair. As recalled in Section 2, this process consists in traversing the torus one dimension after the other, concretely for each dimension δ starting from source vertex δ coordinate and traversing dimension δ until reaching destination vertex δ coordinate. This common algorithm is trivially correct.

For Step 1, it is required to show the existence of a subtorus $T^{\prime }$. Along an arbitrary dimension δ, there are *k* (k>n) subtori $T_{i}$ (0≤i≤k−1). Amongst them, if there is a subtorus $T_{i^{*}}$ that satisfies $|\left(S\cup D\right)\cap T_{i^{*}}|\leq 1$, we can select it as $T^{\prime }$. Now, let us assume that such a subtorus $T_{i^{*}}$ does not exist. Then, because $|\left(S\cup D\right)\cap T_{i}|\geq 2$ (0≤i≤k−1), we have $\sum_{i=0}^{k-1}\left|\left(S\cup D\right)\cap T_{i}\right|\geq 2k>2n$. This induces |S∪D|>2n, which is a contradiction. Hence, $T_{i^{*}}$ always exists.

For Step 2, the existence of pair ρ is trivial. Regarding the existence of a subtorus T⊅ρ, it is recalled that the selection of $T^{\prime }$ fixes dimension δ inducing subtori. Therefore, on the one hand, excluding $T^{\prime }$, there remain k−1 candidate subtori for *T*. On the other hand, selected pair $\rho =(s_{i},d_{i})$ induces at most two additional unavailable subtori for *T* (i.e., if $s_{i},d_{i}$ in the same subtorus, only 1 additional unavailable subtorus is induced, and zero additional unavailable subtorus is induced if $\rho \subset T^{\prime }$). Hence, there remain at least k−3 available subtori for *T*. Since k≥5, we have k−3≥2.

The feasibility of Step 3 is proved by Lemma 2. For Step 4, at most one source-destination pair is to be connected recursively inside $T^{\prime }$. Since n≥3, the dimension of $T^{\prime }$ is at least 2, and the problem can thus be solved recursively in $T^{\prime }$ (this is the base case c=1 of the algorithm). In Steps 4 and 5, the two paths to the appropriate subtorus, say *T*, for a pair $\left(s_{j},d_{j}\right)$ are checked for the inclusion of a common vertex outside *T*. The only case for such a common vertex to exist is when the two paths are in the same direction (i.e., both taken either from $P^{+}$ or $P^{-}$), otherwise this would mean that the common vertex is in *T*. Hence, the common vertex outside *T* that is the closest to $s_{j}$ is also the closest to $d_{j}$.

For Step 6, because one path is connected inside $T^{\prime }$ (or on the way to $T^{\prime }$), the number of paths to select recursively in *T* is at most c−1≤n−1. Since *T* is of dimension n−1, the problem can be solved recursively inside *T*.

Finally, regarding the second base case of the recursion, the special case c=2,n=2, two source-destination pairs need to be connected inside a (2,k)-torus. Each of these two pairs is connected in a distinct subtorus. The existence of the two subtori *T* and $T^{\prime }$ has already been shown previously for the general case.

**Figure 8:**
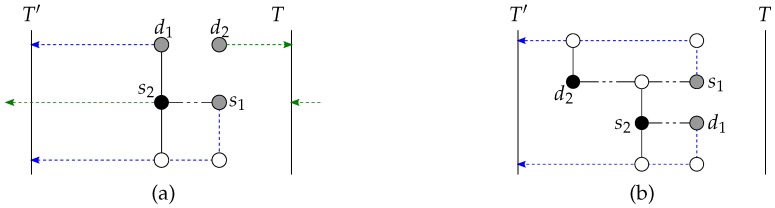
Illustration of the case n=c=2 with the pair $\left(s_{1},d_{1}\right)$ originally selected to be routed to $T^{\prime }$. (**a**) The vertex $s_{2}$ is fully blocked to *T* if not going through $T^{\prime }$. (**b**) Both $s_{2},d_{2}$ are fully blocked to *T*: $\left(s_{2},d_{2}\right)$ is routed instead to $T^{\prime }$, and $\left(s_{1},d_{1}\right)$ to *T*.

The vertices of pair ρ, say $\left(s_{1},d_{1}\right)$, are first routed to $T^{\prime }$ without going through *T*. Since k≥5, there always remains at least one path disjoint with the vertices $\left(s_{2},d_{2}\right)$ of the other pair to route $\left(s_{1},d_{1}\right)$ towards $T^{\prime }$. For the second pair $\left(s_{2},d_{2}\right)$, the paths of $\left(s_{1},d_{1}\right)$ to $T^{\prime }$ may fully block (i.e., not routable as per Lemma 1 to *T* without going through $T^{\prime }$) one or both vertices of $\left(s_{2},d_{2}\right)$. If one single vertex of $\left(s_{2},d_{2}\right)$, say *u*, is fully blocked, route *u* towards *T* by going through $T^{\prime }$ with the path q:p(u,δ) to *T* through $T^{\prime }$. This path *q* always remains unblocked since, for *u* to be fully blocked to *T*, one vertex of $\left(s_{1},d_{1}\right)$, say *v*, is on p(u,δ) to *T*, blocking two candidate paths to *T* for *u*, and the other vertex is in N(u), precisely the neighbor of *u* that is opposite to the neighbor of *u* that is included in the path to $T^{\prime }$ for *v*. Hence, path p(u,δ) to *T* through $T^{\prime }$ is always disjoint with the paths to $T^{\prime }$ selected for $\left(s_{1},d_{1}\right)$. Since with the selection of $s_{2},d_{2}\notin T^{\prime }$, and by the selection of the path *q*, subtorus $T^{\prime }$ includes one single vertex of the path *q*. See Figure 8a. If both vertices of $\left(s_{2},d_{2}\right)$ are fully blocked by the paths to $T^{\prime }$ for $\left(s_{1},d_{1}\right)$, it means that $\left(T\cup T^{\prime }\right)\cap \left(S\cup D\right)=\emptyset$ and that $s_{2},d_{2}$ are blocking both $p(s_{1})$ and $p(d_{1})$ towards *T*. Hence, route instead $\left(s_{2},d_{2}\right)$ to $T^{\prime }$ (with $p\left(s_{2},\delta \right),p\left(d_{2},\delta \right)$ to $T^{\prime }$) and $\left(s_{1},d_{1}\right)$ to *T* (with $p\left(s_{1},\delta \right),p\left(d_{1},\delta \right)$ to *T*). See Figure 8b.

The two subtori $T,T^{\prime }$ are one-dimensional, that is, isomorphic to a ring. It is thus trivial to complete the connection of each of the two pairs inside their respective subtori, even if there is one vertex to be avoided in subtorus $T^{\prime }$: either the clockwise or counter-clockwise ring traversal will do.

## 6. Complexities

Let function T(c,n) represent the time complexity of the proposed algorithm in an (*n*, *k*)-torus with *c* source-destination pairs (c≤n). And, let the function L(c,n) represent the maximum length of a path selected by the algorithm inside an (*n*, *k*)-torus with *c* source-destination pairs (c≤n).

The base case c=1 of the recursion induces the selection of a path with a dimension-order routing algorithm, thus inducing an O(nk) time complexity and a maximum path length of n⌊k/2⌋. Hence, T(1,n)=O(nk) and L(1,n)=n⌊k/2⌋.

For Step 1, subtorus $T^{\prime }$ is selected. A subtorus is selectable as $T^{\prime }$ if the stated three conditions hold. The first condition can be checked by iterating the source and destination vertices, testing inclusion inside the current subtorus. Testing vertex inclusion in a subtorus is achieved by checking the vertex coordinate for each dimension, inducing then an O(n) time complexity. Hence, the first condition is checked for one subtorus in $O(nc^{2})$ time. Checking the second and third conditions induces the same time complexity. Therefore, since for an arbitrary dimension δ at most *c* subtori are unavailable as $T^{\prime }$, it is needed to check at most *c* subtori for the three conditions, thus inducing an $O(nc^{3})$ total time complexity.

For Step 2, selecting the pair ρ is done by iterating each source-destination pair, each time testing the inclusion of the pair source and destination vertices inside $T^{\prime }$. A total time complexity of O(cn) is thus induced. The time complexity of Step 3 is directly induced by Lemma 2: $O(nc^{2})$. For Step 4, the two paths to $T^{\prime }$ are checked similarly, which thus induces an O(nk) time complexity. In addition, if these two paths do not collide, the pair connection is completed in an (n−1, *k*)-torus, thus inducing an O(nk) time complexity. For Step 5, the two paths to *T* for each pair are checked for inclusion of a common vertex. The paths for one pair are thus checked in O(nk) time. Hence, all pair paths are checked in O(cnk) time. For Step 6, time complexity is T(c−1,n−1).

In the special case n=2,c=2, two subtori $T,T^{\prime }$ are selected as in Step 1 of the general case, thus inducing an O(1) time complexity. The selection of the paths for the two pairs may require checking all the candidate paths as per Lemma 1, thus inducing an O(n) time complexity. An additional O(nk) is required to check whether the selected pair paths are disjoint. Finally, routing inside one-dimensional subtori is possible, checking whether the selected path includes the vertex to avoid (O(1)), thus being O(k) time. Hence, in total, this special case is O(nk) time. The maximum path length is obtained when *k* links are taken to route both the source and destination vertices to the designated subtorus, and with k−1 links for routing inside one-dimensional subtori, thus in total 3k−1.

The above discussion can be summarized in the following theorem.

**Theorem** **1.** *In an (n, k)-torus (n<k, k≥5), given c (1≤c≤n) vertex pairs $\left(s_{i},d_{i}\right)$ (all pair vertices are distinct, yet $s_{i}=d_{i}$ is acceptable), it is possible to select c mutually vertex-disjoint paths $s_{i}⇝d_{i}$ (1≤i≤c) of lengths at most 2k(c−1)+n⌊k/2⌋ in $O(nc^{4})$ time.*

**Proof.**  A path of maximum length could be selected as follows: each recursive step induces *k* links to route the source vertex to the designated subtorus, *k* links to route the destination vertex to the designated subtorus, and this until reaching a base case, either c=1 with a path of n⌊k/2⌋ links selected by dimension-order routing, or n=2,c=2 with two paths each of length at most 3k−1. This is summarized with the following recursive expression:

$$\begin{array}{cc} L(1,n) & =n\lfloor k/2\rfloor .\\ L(2,2) & =3k-1.\\ L(c,n) & =2k+L(c-1,n-1). \end{array}$$

Hence, the maximum path length is max{2k(c−1)+n⌊k/2⌋,2k(c−2)+(3k−1)}, which is equal to 2k(c−2)+3k−1 if n=1 and to 2k(c−1)+n⌊k/2⌋ otherwise, given that k≥5. However, since in the n=1 case we have, on the one hand, c=1 and, on the other hand, L(1,1)=⌊k/2⌋, the maximum path length can be expressed as 2k(c−1)+n⌊k/2⌋ for any *n*.

The total worst-case time complexity is given by the following recursive expression:

$$\begin{array}{cc} T(1,n) & =O(nk).\\ T(2,2) & =O(nk).\\ T(c,n) & =O\left(nc^{3}\right)+T\left(c-1,n-1\right). \end{array}$$

Hence, the total time complexity of the proposed routing algorithm is $O(nc^{4})$. ☐

## 7. Empirical Evaluation

We have implemented the algorithm proposed in this paper in order to realize its empirical evaluation, and especially to inspect its practical behavior: how the algorithm performs in average. The computer used for this experiment was equipped with an Intel Core i5-7300U CPU (clocked at 2.60 GHz), 8 GB RAM and ran Windows 10 64-bit.

**Figure 9:**
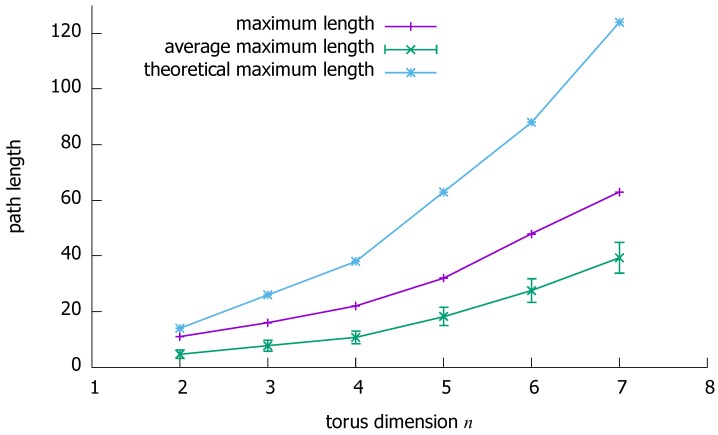
Empirical evaluation: maximum and average maximum (with standard deviation) path lengths of paths selected by the proposed algorithm in an (*n*, max{5,n+1})-torus.

By using this computer implementation, we have repetitively and automatically solved a large number of random instances of the pairwise disjoint-path routing problem in a torus. Precisely, in an (*n*, max{5,n+1})-torus, we have solved for each value of *n* (2≤n≤7) a total of 10,000 random routing problem instances. For each of these instances, the correctness of the selected paths has been checked (e.g., path disjointness), and the maximum length of the selected paths was stored. Then, for each value of *n*, the average of the stored maximum path lengths for this value of *n* was computed. Hence, our results include for each *n* both the maximum and the average of the obtained 10,000 maximum path lengths. The number of vertex pairs was set to c=n to maximize the routing difficulty. For each *n*, the maximum and average of the maximum path lengths are given in Figure 9; the theoretical upper bound on the maximum path length is also given for reference.

**Figure 10:**
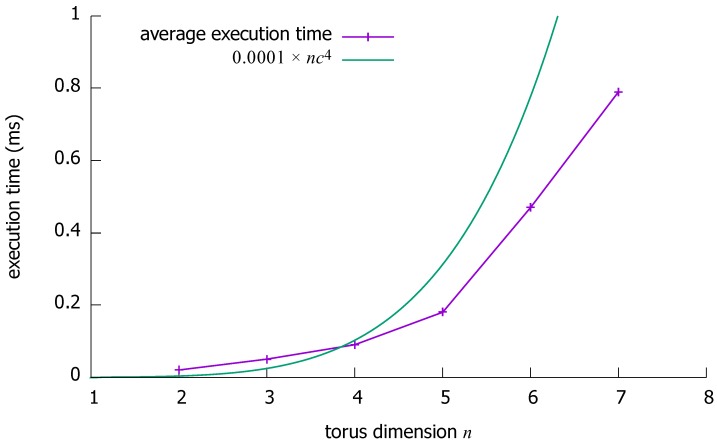
Empirical evaluation: average execution time to solve one problem instance with the proposed algorithm in an (*n*, max{5,n+1})-torus.

Then, we have measured the average execution time for one problem instance: see Figure 10; the worst-case time complexity is also plotted for reference.

Regarding maximum path lengths, one can first note that there is some distance between the theoretical upper bound on the maximum path length and the obtained results. This is a good indicator of the efficiency of the proposed algorithm, especially given that for small values of *n* the upper bound is almost tight on the maximum path length. Regarding time complexity, it can be observed from the obtained results that the average performance of the algorithm is significantly better than the theoretical worst-case time complexity.

## 8. Conclusions

The torus topology is very popular for the interconnection network of massively parallel systems such as the modern supercomputers Fujitsu K, IBM Blue Gene/L, IBM Blue Gene/P, and Cray Titan. In this paper, an algorithm that solves the pairwise disjoint-path routing problem in a torus has been proposed. This routing problem consists in the selection of mutually vertex-disjoint paths between given vertex pairs, and it is critical to maximize system dependability and data communication efficiency. We have then formally shown that in an *n*-dimensional *k*-ary torus, for *c* (c≤n) vertex pairs, the described algorithm selects disjoint paths of lengths at most 2k(c−1)+n⌊k/2⌋ with a worst-case time complexity of $O(nc^{4})$. Furthermore, the practical behavior of the proposed algorithm has been inspected by conducting computer experiments, showing that performance on average was significantly better than the established formal worst-case complexities.

As for future works, it will be meaningful to investigate whether the theoretical maximum path length is attainable, and if not, try to refine it. For example, this might be achieved by more wisely selecting the subtori used for disjoint routing. Even though the selection of subtorus $T^{\prime }$ may not leave many options, the selection of subtorus *T* that was used to solve the problem recursively could be done such that shorter paths to route pair vertices to *T* are selected.

## Figures and Tables

**Table 1:** Comparing torus topological properties with other popular interconnection networks [9].

Network	Order	Degree	Diameter	Cost	Links
(*n*, *k*)-torus	$k^{n}$	2n	n⌊k/2⌋	$2n^{2}\left\lfloor k/2\right\rfloor$	$nk^{n}$
*n*-hypercube	$2^{n}$	*n*	*n*	$n^{2}$	$2^{n}n$
*n*-dual-cube	$2^{2n-1}$	*n*	2n	$2n^{2}$	$2^{2n-2}n$
*n*-star graph	n!	n−1	⌊3(n−1)/2⌋	(n−1)⌊3(n−1)/2⌋	n!(n−1)/2
(*k*, *m*)-metacube	$2^{2^{k}m+k}$	m+k	$2^{k}\left(m+1\right)$	$2^{k}\left(m+1\right)\left(m+k\right)$	$2^{2^{k}m+k-1}\left(m+k\right)$

**Table 2:** A possible algorithm execution trace in a (4, 5)-torus and the four vertex pairs $\{\left(s_{1},d_{1}\right),$$\left(s_{2},d_{2}\right),\left(s_{3},d_{3}\right),\left(s_{4},d_{4}\right)\}$ where $S=\{s_{1}=\left(2,1,0,4\right),s_{2}=\left(0,2,1,2\right),s_{3}=\left(2,4,0,2\right),s_{4}=\left(4,4,4,1\right)\}$ and $D=\{d_{1}=\left(0,0,4,4\right),d_{2}=\left(3,2,0,2\right),d_{3}=\left(0,4,0,3\right),d_{4}=\left(0,4,0,2\right)\}$.

n	**Pairs**	δ	$\gamma (T^{\prime },\delta )$	γ(T,δ)	**Selected paths to $T^{\prime }$**
4	$(s_{1},d_{1}),$ $(s_{2},d_{2}),$ $(s_{3},d_{3}),$ $\left(s_{4},d_{4}\right)$	1	1	3	$s_{1}\to \left(1,1,0,4\right)=s_{1}^{\prime }$
					$d_{1}\to \left(1,0,4,4\right)=d_{1}^{\prime }$
					**Selected paths to T**
					$s_{4}\to \left(3,4,4,1\right)=s_{4}^{\prime }$
					$d_{4}\to \left(0,3,0,2\right)\to \left(4,3,0,2\right)\to \left(3,3,0,2\right)=d_{4}^{\prime }$
					$s_{3}\to \left(3,4,0,2\right)=s_{3}^{\prime }$
					$d_{3}\to \left(4,4,0,3\right)\to \left(3,4,0,3\right)=d_{3}^{\prime }$
					$s_{2}\to \left(4,2,1,2\right)\to \left(3,2,1,2\right)=s_{2}^{\prime }$
					$d_{2}$
Path completion in $T^{\prime }$: $s_{1}^{\prime }\to \left(1,0,0,4\right)\to d_{1}^{\prime }$.					
n	**Pairs**	δ	$\gamma (T^{\prime },\delta )$	γ(T,δ)	**Selected paths to $T^{\prime }$**
3	$(s_{2}^{\prime },d_{2}),$ $(s_{3}^{\prime },d_{3}^{\prime }),$ $\left(s_{4}^{\prime },d_{4}^{\prime }\right)$	2	0	1	$s_{2}^{\prime }\to \left(3,3,1,2\right)\to \left(3,4,1,2\right)\to \left(3,0,1,2\right)=s_{2}^{\prime \prime }$
					$d_{2}\to \left(3,2,4,2\right)\to \left(3,3,4,2\right)\to \left(3,4,4,2\right)\to \left(3,0,4,2\right)=d_{2}^{\prime }$
					**Selected paths to T**
					$s_{3}^{\prime }\to \left(3,4,0,3\right)\to \left(3,3,0,3\right)\to \left(3,2,0,3\right)\to \left(3,1,0,3\right)=s_{3}^{\prime \prime }$
					$d_{3}^{\prime }\to \left(3,3,0,3\right)\to \left(3,2,0,3\right)\to \left(3,1,0,3\right)=d_{3}^{\prime \prime }$
					$s_{4}^{\prime }\to \left(3,3,4,1\right)\to \left(3,2,4,1\right)\to \left(3,1,4,1\right)=s_{4}^{\prime \prime }$
					$d_{4}^{\prime }\to \left(3,3,0,1\right)\to \left(3,2,0,1\right)\to \left(3,1,0,1\right)=d_{4}^{\prime \prime }$
Path completion in $T^{\prime }$: $s_{2}^{\prime \prime }\to \left(3,0,0,2\right)\to d_{2}^{\prime }$, and en route to *T*: $s_{3}^{\prime }\to d_{3}^{\prime }$.					
n	**Pairs**	**Selected path**			
2	$\left(s_{4}^{\prime \prime },d_{4}^{\prime \prime }\right)$	$s_{4}^{\prime \prime }\to d_{4}^{\prime \prime }$ (base case c=1: dimension-order routing)			

## Appendix A. Additional Details of Candidate Paths

In an (*n*, *k*)-torus, given vertex $u=(u_{1},u_{2},\ldots ,u_{n})$ and dimension δ (1≤δ≤n), path $p^{-}\left(u,\delta \right)$ and path sets $P_{1}^{-}\left(u,\delta \right)$, $P_{2}^{-}\left(u,\delta \right)$ are defined similarly to $p^{+}\left(u,\delta \right)$, $P_{1}^{+}\left(u,\delta \right)$, and $P_{2}^{+}\left(u,\delta \right)$, respectively, as described in Section 2. The formal definitions of the path $p^{-}\left(u,\delta \right)$ and the path sets $P_{1}^{-}\left(u,\delta \right)$, $P_{2}^{-}\left(u,\delta \right)$ are given below.

$$p^{-}\left(u,\delta \right):\;u\to u⊖e_{\delta }\to \left(u⊖e_{\delta }\right)⊖e_{\delta }\to \ldots$$

$$\begin{array}{c} P_{1}^{-}\left(u,\delta \right):\;{⋃}_{\begin{array}{c} 1\leq i\leq n\\ i\neq \delta \end{array}}\left\{\begin{array}{c} u\to u⊕e_{i}\to \left(u⊕e_{i}\right)⊖e_{\delta }\to \left(\left(u⊕e_{i}\right)⊖e_{\delta }\right)⊖e_{\delta }\to \ldots ,\\ u\to u⊖e_{i}\to \left(u⊖e_{i}\right)⊖e_{\delta }\to \left(\left(u⊖e_{i}\right)⊖e_{\delta }\right)⊖e_{\delta }\to \ldots \end{array}\right\} \end{array}$$

$$\begin{array}{c} P_{2}^{-}\left(u,\delta \right):\;{⋃}_{\begin{array}{c} 1\leq i\leq n\\ i\neq \delta \end{array}}\left\{\begin{array}{c} u\to u⊕e_{i}\to \left(u⊕e_{i}\right)⊕e_{i}\to \left(\left(u⊕e_{i}\right)⊕e_{i}\right)⊖e_{\delta }\\ \;\to \left(\left(\left(u⊕e_{i}\right)⊕e_{i}\right)⊖e_{\delta }\right)⊖e_{\delta }\to \ldots ,\\ u\to u⊖e_{i}\to \left(u⊖e_{i}\right)⊖e_{i}\to \left(\left(u⊖e_{i}\right)⊖e_{i}\right)⊖e_{\delta }\\ \;\to \left(\left(\left(u⊖e_{i}\right)⊖e_{i}\right)⊖e_{\delta }\right)⊖e_{\delta }\to \ldots \end{array}\right\} \end{array}$$
